# LAPAROSCOPIC DISTAL PANCREATECTOMY WITH SPLEEN PRESERVATION

**DOI:** 10.1590/0102-672020180001e1395

**Published:** 2018-08-16

**Authors:** Sergio Renato PAIS-COSTA, Guilherme Costa Crispim de SOUSA, Sergio Luiz Melo ARAUJO, Olímpia Alves Teixeira LIMA

**Affiliations:** 1 Hospital Santa Lúcia, Brasília, DF; 2 Hospital Brasília, Brasília, DF, Brasil.

**Keywords:** Pancreatectomy, Laparoscopy, Surgery, Pancreatic neoplasms, Pancreatectomia, Laparoscopia, Cirurgia, Neoplasias pancreáticas

## Abstract

**Background:**

Laparoscopic distal pancreatectomy has been the choice for resection of distal pancreas lesions due many advantages over open approach. Spleen preservation technique seems minimizes infectious complications in long-term outcome.

**Aim:**

To present the results of laparoscopic distal pancreatectomies with spleen preservation by Kimura´s technique (preservation of spleen blood vessels) performed by single surgical team.

**Methods:**

Retrospective case series aiming to evaluate both short and long-term outcomes of laparoscopic distal pancreatectomies with spleen preservation.

**Results:**

A total of 54 laparoscopic distal pancreatectomies were performed, in which 26 were laparoscopic distal pancreatectomies with spleen preservation by Kimura´s technique. Mean age was 47.9 years-old (21-75) where 61.5% were female. Mean BMI was 28.5 kg/m² (18-38.8). Mean diameter of lesion was 4.3 cm (1.8-7.5). Mean operative time was 144.1 min (90-200). Intraoperative bleeding was 119.2 ml (50-600). Conversion to laparotomy 3% (n=1). Postoperative morbidity was 11.5%. Postoperative mortality was null. Mean of hospital stay was 4.8 days (2-14). Mean time of follow-up period was 19.7 months (2-60). There was no neoplasm recurrence or mortality on evaluated period. There was no infectious complication.

**Conclusion:**

Laparoscopic distal pancreatectomy with spleen and splenic vessels preservation is feasible, safe, and effective procedure. This technique presented both low morbidity and null mortality on this sample. There were neither infectious complications nor neoplasm recurrence on long-term follow-up period.

## RESUMO

**Racional:**

A pancreatectomia distal laparoscópica tem sido a abordagem de escolha para ressecção de tumores em pâncreas distal devido a muitas vantagens sobre a abordagem laparotômica. A técnica com preservação esplênica parece minimizar complicações infecciosas em longo prazo.

**Objetivo:**

Analisar os resultados das pancreatectomias distais laparoscópicas com preservação esplênica e dos vasos esplênicos.

**Método:**

Série de casos com 26 doentes operados para avaliar os resultados em curto e longo prazo.

**Resultados:**

Cinquenta e quatro pancreatectomias distais laparoscópicas foram realizadas nesse período, onde 26 foram pancreatectomias distais laparoscópicas com preservação esplênica pela técnica de Kimura. A média de idade foi 47,9 anos (21-75), onde 61,5% eram mulheres. A média do IMC foi 28,5 kg/m² (18-38,8). O diâmetro médio das lesões foi 4,3 cm (1,8-7,5). O tempo cirúrgico médio foi 144,1 min (90-200). A média de sangramento intra-operatório foi 119,2 ml (50-600). O índice de conversão foi 3% (n=1). A morbidade pós-operatória foi 11,5%, e a mortalidade nula. A média de internação hospitalar foi 4,8 dias (2-14). A média de seguimento foi 19,7 meses (2-60). Não houve recorrência de neoplasias e nem mortalidade durante esse período e também não houve complicações infecciosas em longo prazo.

**Conclusão:**

A pancreatectomia distal laparoscópica com preservação esplênica e dos vasos esplênicos é procedimento factível, seguro e eficaz no tratamento de neoplasias pancreáticas. Esta técnica apresentou baixa morbidade e mortalidade. Não houve complicações infecciosas e nem recidiva neoplásica em longo prazo.

## INTRODUCTION

Laparoscopy has now been introduced as a therapeutic option for treating the majority of digestive system diseases and for other surgical areas, including for more complex procedures, such as hepatobiliopancreatic surgery. Laparoscopic pancreatic surgery began with resection of small and benign lesions (enucleation) and progressed to caudal, total, central and duodenal pancreatectomy procedures^3^ ^,^ ^11^ ^,^ ^12^ ^,^ ^19^. Despite all the technical difficulties inherent to pancreatic surgery, laparoscopic distal pancreatectomy has been gradually included in the routine of various services because their results are similar to those from laparotomy, including in cases of malignant disease^8^ ^,^ ^18^.

Various forms of distal pancreatic disease with surgical indications can be treated via laparoscopy. This may be performed with or without splenectomy, depending on the nature of the lesion (benign or malignant), histological type (neuroendocrine tumors can be removed with preservation of the spleen) and any presence of local invasion^14^ ^,^ ^15^. 

In cases of larger tumors or those that present significant local invasions, laparoscopy is normally only used for intraoperative staging. These procedures are then converted into laparotomy for multiple visceral resections when there is no peritoneal carcinomatosis. Therefore, preoperative staging is paramount for defining the surgical route.

The objective of this study was to present the results from all laparoscopic distal pancreatectomy cases with preservation of the spleen conducted over an eight-year period. 

## METHODS

This is a retrospective series of laparoscopic distal pancreatectomies performed between January 2009 and September 2016 at Hospital Santa Lúcia and Hospital Brasília, in Brasília, DF, Brazil. A specific protocol was drawn up for following up all cases of distal pancreatic tumors that underwent distal pancreatectomy with and without splenectomy, with the objective of evaluating short-term results (duration of surgery, bleeding, transfusion, conversion rate, morbidity, mortality and histopathological analysis) and long-term results (recurrence, survival and late complications) from these procedures.

**FIGURE 1:**
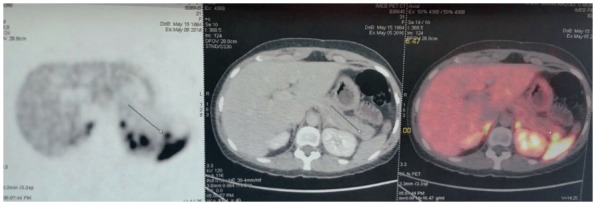
Octreotide scintigraphy showing a pancreatic tail neuroendocrine tumor

All the patients underwent preoperative evaluation, using both contrast-enhanced thin-slice computed tomography for the pancreas and nuclear magnetic resonance for estimating lesion size and loco-regional or distant staging. Some cases also underwent endoscopic ultrasound with biopsy in order to define the type of lesion and surgical indication. Neuroendocrine tumors were also staged using octreotide scintigraphy (octreo-scan, Figure 1). 

### Surgical technique

The surgical technique was standardized by the team for all procedures. The patient was positioned in dorsal decubitus, slightly inclined, with legs apart and the upper body lateralized to the right. The surgeon took up a position between the patient’s legs, with the first assistant to the right, and the second assistant to the left (optics). Five trocars were used and the procedure began with freeing of the greater omentum from the greater curvature of the stomach. The stomach was then fixed to the abdominal wall by the greater curvature and the short gastric vessels were freed to enable proper viewing of the omental bursa. After dissection of the posterior face of the pancreas, the splenic artery and vein were dissected and isolated, and were then repaired by means of a vessel loop or cardiac tape. 

**FIGURE 2:**
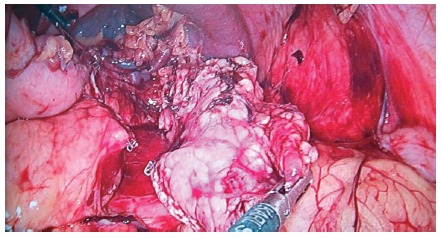
Sectioning the pancreatic tail after isolation and preservation of splenic vessels

**FIGURE 3:**
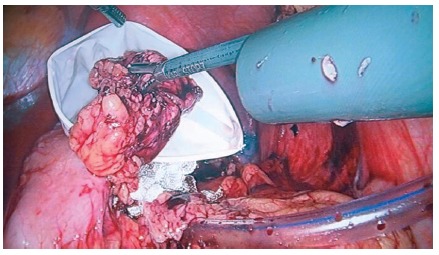
Removal of specimen and cavity drainage

In cases of preservation of the spleen, the technique proposed by Kimura et al^7^ was used. In this, the vessels were released by means of ligature of small branches, of both the splenic vein and the splenic artery, using ultrasonic energy or bipolar tweezers (Ultracision, Ethicon or Sonicision, Covidien), and metal or Hem-o-lok clips. Proximal pancreatic body stapling was performed using a 30 or 45 mm vascular load or thick-tissue stapler (Endoguia, Covidien or Echelon-Ethicon) depending on tissue texture (Figure 2). Posterior pancreatic dissection was done on the splenic vessels as far as their hilum. After complete release of the specimen containing the body and tail of the pancreas, it was removed, protected in an Endobag or glove (Figure 3), preferentially through a small incision after expansion of the site where the 12-mm trocar was positioned in the left flank, in the case of small lesions. More rarely, in the case of larger specimens, removal was done through a Pfannenstiel transverse incision in the lower abdomen. After reviewing the hemostasis, fibrin glue was applied to the stump of the pancreas and a tubular drain was positioned. 

During the immediate postoperative period, all patients were sent to the intensive care unit. Tubular drain debit was measured daily and the output was sent to the laboratory to perform an amylase assay on the third day. If the amylase level was more than three times higher than its serum level, a situation of pancreatic fistula was diagnosed. These fistulas were classified in accordance with the ISGPF standard (International Study Group of Pancreatic Fistula), and treatment was individualized for each case.

## RESULTS

A total of 54 laparoscopic distal pancreatectomies were performed. In this sample, there were 28 cases of distal pancreatectomies with splenectomy and 26 with preservation of the spleen. Patients who underwent splenectomy in association with pancreatectomy were excluded. 

The mean age of the 26 patients with preservation of the spleen was 47.9 years (range: 21-75). There were 16 women and 10 men, with a mean BMI of 28.5 kg/m² (range: 18-38.8). Among the comorbidities, five patients were hypertensive and three were diabetic. The mean lesion size was 4.3 cm (range: 1.8-7.5).

The mean volume of bleeding was 119.2 ml (range: 50-600) and the mean duration of the surgical procedure was 144.1 min (range: 90-200). Blood transfusion was only necessary in one patient. The mean length of hospital stay was 4.8 days (range: 2-14). The histopathological analysis showed negative margins in all cases. In one of the procedures, splenectomy had to be performed and conversion into laparotomy was performed because of significant bleeding. No patient died and no reoperation was required. The overall morbidity rate was 11.5%. Four pancreatic fistulas were observed during the postoperative period: one of grade A and three of grade B. The grade-A fistula was treated conservatively, and all the grade-B fistulas were treated by means of puncture: two cases of percutaneous puncture, and one of echoendoscopy. Among the late complications, only one patient presented segmental splenic infarction, which was diagnosed from a follow-up imaging examination. However, no clinical repercussion was observed and it was decided simply to monitor the condition: this patient presented good evolution. No patient presented any endocrine or exocrine insufficiency in this sample. The mean length of follow-up was 19.7 months (range: 2-60 months) and, within this period, no recurrence was observed. These characteristics are shown in Table 1 and the histological types of the various cases in Table 2. 

**TABLE 1:** Early and late results (n=26)

Age	47.9 years (21 - 75)
Sex	
Female	16 (61.5%)
Male	10 (38.5%)
BMI	28.5 kg/m² (18 - 38.8)
Lesion size	4.3 cm (1.8 - 7.5)
Surgery duration	144.1 minutes (90 - 200)
Bleeding	119.2 ml (50 - 600)
Hospital stay	4.8 days (2 - 14)
Conversion	1 (3.8%)
Mortality	0
Pancreatic fistula (grades B and C)	3 (11.5%)
Follow-up	19.7 months (2 - 60)
Recurrence	0

**TABLE 2:** Histological types of the lesions

Histological type	n	%
Mucinous cystadenoma	7	26.9
Serous cystadenoma	7	26.9
IPMN	5	19.2
Neuroendocrine tumor	4	15.4
SCPT (Frantz)	2	7.7
Accessory spleen	1	3.8

IPMN=intraductal papillary mucinous neoplasia; SCPT=solid-cystic pseudopapillary tumor (Frantz)

## DISCUSSION

Laparoscopic distal pancreatectomy has been successfully performed by several authors. Large tumors or tumors with invasion of adjacent organs for which there is a surgical indication undergo open distal pancreatectomy. Within this eight-year period, 54 laparoscopic surgical procedures were performed, among which 28 were associated with splenectomy, and 26 presented preservation of the spleen. Although preservation of the spleen is a surgical technique for which surgeons require greater experience and knowledge, it provides well-established benefits to patients through reducing infectious complications, morbidity, length of hospital stay and post-splenectomy sepsis, and therefore should be performed whenever possible^4^ ^,^ ^17^. In addition, the spleen presents an essential function in the immunological system, by promoting protection against encapsulated bacterial infections.

Preservation of the spleen can be performed by using two surgical techniques. The one performed in all cases of the present study involved preservation of splenic vessels from their origin to the hepatic hilum (technique proposed by Kimura et al^7^). The other technique, described by Warshaw et al^20^, in which splenic vessels are ligated at their origin, maintains splenic irrigation only through short gastric vessels. The present team chose, whenever possible, to avoid using the Warshaw technique because of the increased risk of splenic infarction and the need to perform splenectomy subsequently^13^. In addition, this technique seems to predispose patients to higher incidence of gastric varices during the late postoperative period^5^.

Only a few case series of patients who underwent laparoscopic distal pancreatectomies with preservation of the spleen through use of the technique proposed by Kimura have been described in the literature, and the majority have had samples of fewer than 10 patients^1^ ^,^ ^2^ ^,^ ^21^ ^,^ ^22^. In most studies, distal pancreatectomy was performed with splenectomy or with preservation of the spleen using the technique proposed by Warshaw et al.^20^ because these are technically simpler procedures. Among the studies that used the technique proposed by Kimura et al^7^, the main ones have been selected for presentation in Table 3.

**TABLE 3:** Results from other studies on distal pancreatectomy with laparoscopic preservation of the spleen

	LDPSP	Lesion size	Surgery duration	Bleeding	Pancreatic fistula (B and C)	Hospital stay	Mortality
Adam et al^1^, 2013	55	3.3 cm	214.7 min	342.8 ml	3.60%	8.2 days	0%
Beane et al^2^, 2010	45	NA	NA	224 ml	2%	4.5 days	0%
Worhunsky et al^21^, 2014	19	1.6 cm	201 min	40 ml	6%	3.3 days	0%
Yan et al^22^, 2014	38	4.5 cm	123 min	78.2 ml	5.30%	7.6 days	0%
Present study	26	4.3 cm	144.1 min	119.2 ml	11.50%	4.8 days	0%

NA=not available; LDPSP=laparoscopic distal pancreatectomy with spleen preservation

In Brazil, as far as the present authors know, this is the first study conducted exclusively on a series of cases of laparoscopic distal pancreatectomy with preservation of the spleen using the technique proposed by Kimura et al^7^ (preservation of splenic vessels). Machado et al^9^ described a series of cases of laparoscopic pancreatic procedures, in which 12 distal pancreatectomy procedures with preservation of the spleen were performed, although without specifying the technique used or the specific results from this subgroup.

The present team showed that the duration of surgery, blood loss and length of hospital stay were similar to the findings from other studies. Moreover, there were no deaths in this sample. This shows that this procedure is also possible, safe and effective in our environment. 

Among the procedures performed, none of the patients needed reoperation or presented any significant early or late infectious complications. Among the late complications, only one patient presented segmental splenic infarction, which was diagnosed from a follow-up imaging examination. This condition did not have any clinical repercussion and expectant monitoring was approach chosen.

The pancreatic fistula rate was greater in this sample than in the other studies. This finding can be explained by the high BMI presented by our patients, because this factor poses a high risk of pancreatic fistula formation, according to some studies^6^ ^,^ ^16^. Another possible explanation relates to the closure of the pancreatic stump, which was performed through using a linear stapler and fibrin glue. However, the method to be used for this closure is a matter of controversy, without any consensus regarding the best method to be used^10^.

Among the lesions that underwent resection using this technique, the histopathological evaluation only showed low-grade malignancy. These lesions did not require lymphadenectomy of the splenic bed, which allowed safe preservation of the spleen. For larger lesions or lesions that were known to present high risk of recurrence, such as adenocarcinoma, the present team performed pancreatectomy with splenectomy via laparoscopy (whenever possible) or laparotomy procedures.

No cases of recurrence of neoplasia were observed in this group of patients during the follow-up, due probably both to the small lesion size and to their low degree of malignancy. Nor were there any long-term infectious complications caused by encapsulated bacteria.

Although this study may present several limitations, such as various forms of disease and selection bias (since it was decided only to include selected cases for this technique, such as minor tumors without significant local invasion, and those with low degrees of malignancy), our opinion is that it presents value in that its data can possibly be integrated into larger studies with broader samples. Another study (in progress) is being conducted by the present team in which pancreatectomy procedures with splenectomy will be compared with pancreatectomies with preservation of the spleen (preservation of splenic vessels). There are only a few comparative studies analyzing both techniques, especially including the method proposed by Kimura et al.^7^

## CONCLUSION

Laparoscopic distal pancreatectomy with preservation of the spleen through using the technique proposed by Kimura, with preservation of splenic vessels, is a feasible, safe and effective procedure for treating small pancreatic neoplasms with low-grade malignancy. This technique presented low morbidity and zero mortality. No infectious complications or recurrences of neoplasia were observed over the study period. 
